# A preliminary study on the application of deep learning methods based on convolutional network to the pathological diagnosis of PJI

**DOI:** 10.1186/s42836-022-00145-4

**Published:** 2022-10-14

**Authors:** Ye Tao, Hanwen Hu, Jie Li, Mengting Li, Qingyuan Zheng, Guoqiang Zhang, Ming Ni

**Affiliations:** Department of Orthopedics, the Fourth Medical Center, Chinese PLA General Hospital, 51 Fucheng Road, Beijing, 100036 China

**Keywords:** PJI (Prosthetic Joint Infections), Deep learning, Infected area, Neutrophil count

## Abstract

**Objective:**

This study aimed to establish a deep learning method based on convolutional networks for the preliminary study of the pathological diagnosis of prosthetic joint infections (PJI).

**Methods:**

We enrolled 20 revision patients after joint replacement from the Department of Orthopedics, the First Medical Center, General Hospital of the People's Liberation Army, from January 2021 to January 2022 (10 of whom were confirmed to be infected against 2018 ICM criteria, and the remaining 10 were verified to be non-infected), and classified high-power field images according to 2018 ICM criteria. Then, we inputted 576 positive images and 576 negative images into a neural network by employing a resNET model, used to select 461 positive images and 461 negative images as training sets, 57 positive images and 31 negative images as internal verification sets, 115 positive images and 115 negative images as external test sets.

**Results:**

The resNET model classification was used to analyze the pathological sections of PJI patients under high magnification fields. The results of internal validation set showed a positive accuracy of 96.49%, a negative accuracy of 87.09%, an average accuracy of 93.22%, an average recall rate 96.49%, and an F1 of 0.9482. The accuracy of external test results was 97.39% positive, 93.04% negative, the average accuracy of external test set was 93.33%, the average recall rate was 97.39%, with an F1 of 0.9482. The AUC area of the intelligent image-reading diagnosis system was 0.8136.

**Conclusions:**

This study used the convolutional neural network deep learning to identify high-magnification images from pathological sections of soft tissues around joints, against the diagnostic criteria for acute infection, and a high precision and a high recall rate were accomplished. The results of this technique confirmed that better results could be achieved by comparing the new method with the standard strategies in terms of diagnostic accuracy. Continuous upgrading of extended training sets is needed to improve the diagnostic accuracy of the convolutional network deep learning before it is applied to clinical practice.

## Background

Periprosthetic joint infection (PJI) represents a devastating complication after arthroplasty [1]. As the third most common cause of failure in primary joint replacement surgery and the most common cause of failure in revision surgery, PJI has attracted special attention of joint surgeons. According to literature, poor understanding of low-grade infection [2–5] and missed diagnosis tended to result in inappropriate treatment. For example, non-infectious treatment measures might be in appropriately used for patients with periprosthetic infection. Although orthopedists and scientists have endeavored to avoid PJI, across the globe, the overall incidence after joint replacement has not shown a substantial decline in the past years [6].

As we know it, effective treatment starts with accurate diagnosis. Since the introduction of low friction arthroplasty in 1961, researchers have been struggling to figure out the way to accurately diagnose PJI [7]. In 2011, Musculoskeletal Infection Society (MSIS) proposed diagnostic criteria for PJI on the basis of clinical symptoms, serological results and histopathological findings [8, 9]. In 2018, International Consensus Conference (ICM) put forward a new definition of PJI [10] upon long-term cohort studies and literature review [3], and modified the weighted scores of various diagnostic criteria. These diagnostic criteria integrated multiple indicators covering serology, pathology, gene sequencing and microbiology, among others. Against the novel criteria, the diagnosis of PJI based on a single indicator is often considered inadequate. Over the past decades, neutrophil count has been employed for the pathological diagnosis for its high specificity [11].

Many challenges remain in the traditional diagnosis of local infection of joint synovial soft tissues. According to the current ICM consensus in 2018, if more than 10 neutrophils are found in a 400 × high magnification field, the field is deemed positive for infection [10]. However, neutrophils vary morphologically and experience is needed in the pathological identification of infection. On the other hand, the manual scanning of the entire section is difficult and recording the number of neutrophils with different morphologies presents another challenge. These difficulties impair the accuracy and sensitivity of pathological diagnosis [12]. Subjective selection of target areas leads to poor repeatability/reproducibility and reduces the power of intraoperative pathological evidence. In addition, the time-consuming intraoperative frozen section procedure can’t satisfy the timeliness as required by the relevant guidelines.

The twentieth century witnessed the appearance of machine learning and scientists have been employing machine learning as a research tool to resolve problems that are humanly impossible to address, leading to the advent of artificial intelligence technology. At present, with the rapid development of artificial neural network, artificial intelligence technology has been applied in the medical and health fields, and its application in medicine has brought profound changes to the medical practice and researches. With the incremental progress of the artificial intelligence technology, it has been increasingly used in the various fields of medical sciences, including natural language processing, speech recognition, diagnosis aided by computerized visioning, image recognition, big data analysis, pharmaceutic research and development. Astuto et al. [13] developed an automatic model based on knee joint data using CNN, which can automatically detect and grade injuries of bone, cartilage, meniscus and ACL. Meanwhile, deep learning has been applied to automatic segmentation and classification of colonoscopically-collected tissues, thus allowing for automatic analysis of colorectal cancer specimens [14, 15]. In addition, the deep-learning-based segmentation and classification of glomeruli with different pathological changes shows good prospect of application in the field of nephrology [16].

The purpose of this study was to establish a model for the pathological diagnosis of PJI by employing a deep learning method based on convolutional network. We, by computerized classification of the HE-stained specimens of tissues in the adjacent of infected joint, assessed whether the specimens were infected and the result was used as evidence for the diagnosis of PJI. Moreover, we preliminarily explored the use of multiple infection indicators for the diagnosis of PJI under high-magnification fields.

## Methods

### Material acquisition

This study enrolled 20 revision patients after joint replacement from the Department of Orthopedics of the First Medical Center of Chinese PLA General Hospital, from January 2021 to January 2022. Among them, 10 patients were diagnosed with PJI and 10 were non-infected patients, according to the 2018 ICM Diagnostic Guidelines. The basic data of the patients are listed in Table 4. All the 10 patients with confirmed PJI met one of the main diagnostic criteria in the 2018 PJI Diagnostic Guidelines and culture revealed bacteria in both the joint fluid and tissue masses around the prosthesis. The bacteria identified and serological indicators are given in Table 5. Tissue samples were harvested from the patients during surgery for the preparation of frozen sections. The sample collection was done in strict accordance with the 2018 ICM diagnostic criteria. ICM diagnostic criteria recommended intraoperative collection of specimens at three or more sites of suspected soft tissue infection. In this study, if knee infection was suspected, samples were taken at the distal part of femur, proximal end of tibia and from joint capsule. In cases suspected of hip infection, the samples were harvested near the femoral head, acetabula, and from joint capsule. According to the criteria, the tissue samples collected were bagged separately and used as the tissue source for the pathological diagnosis. Intraoperatively, the tissue samples were collected by a doctor from the Pathology Department of the center and were subjected to routine HE staining.

### Model establishment

**Fig. 1:**
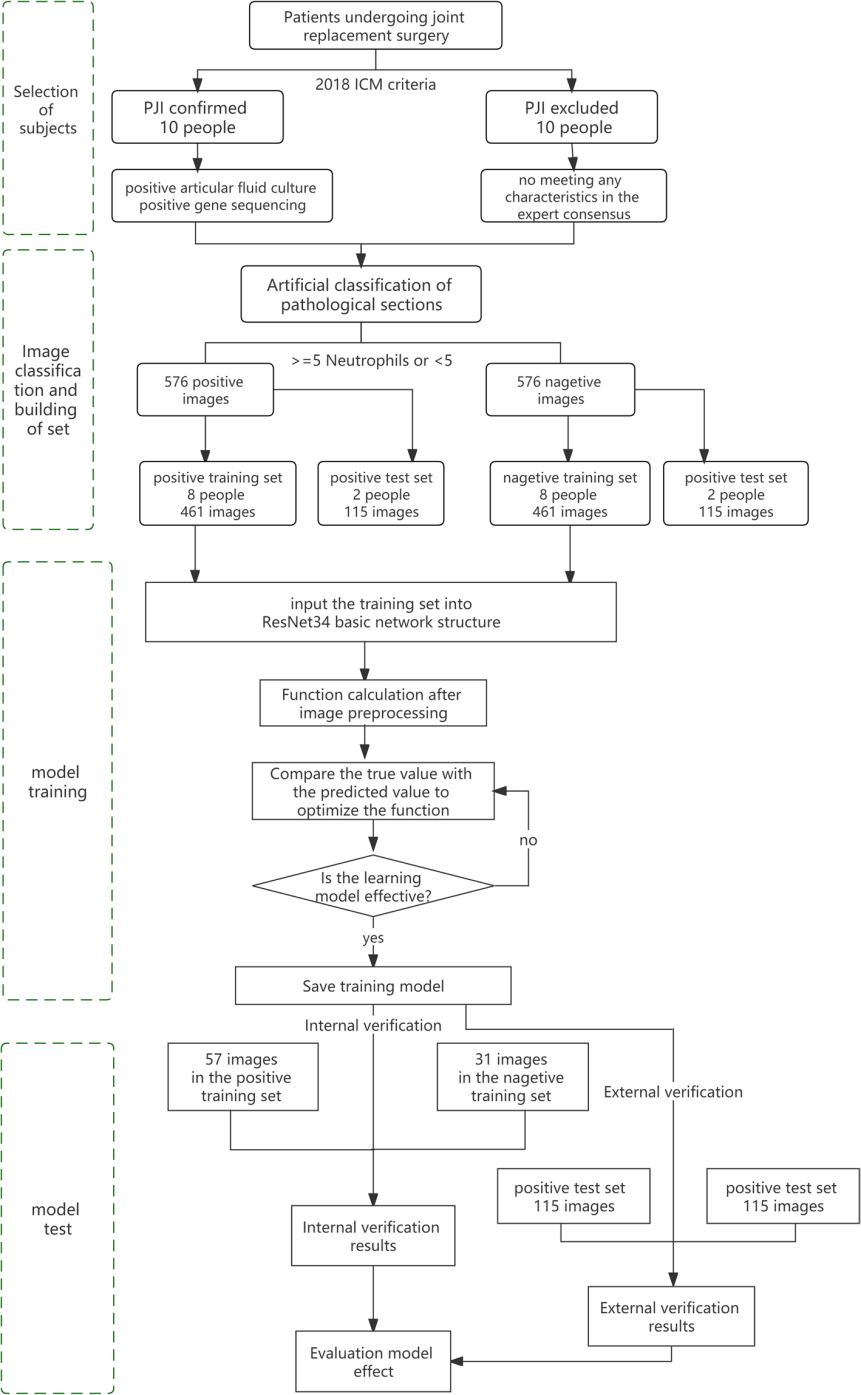
Overall flow of experiment

**Fig. 2:**
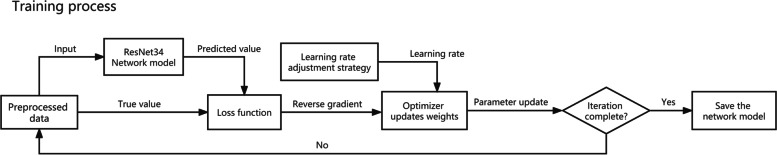
Process of Training

**Fig. 3:**
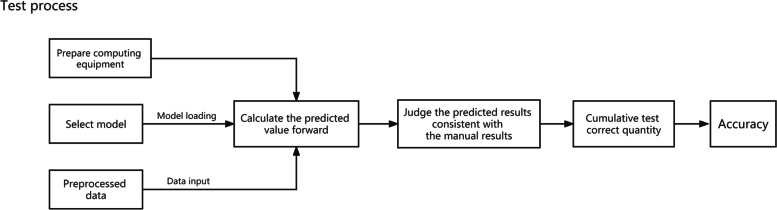
Process of test

The frozen pathological sections of the above 20 patients were converted into electronic images by using UN scanner and McUddy scanner, and then the entire section (image) was artificially segmented under the high magnifications (400 ×) and observed by employing IViewer software package. The segmented electronic images were saved in TIFF format. Two pathologists read the images and made pathological diagnosis against the 2018 ICM diagnostic criteria. According to the criteria, a white blood cell count greater than 10 under high magnifications is deemed positive, otherwise negative. A total of 576 positive images (from 10 patients diagnosed with PJI) and 576 negative ones (from 10 uninfected patients) were identified. Eight positive patients (including 461 images) and 8 negative patients (involving 461 images) were included in a training set, and the remaining two positive patients (115 images) and two negative patients (115 images) were assigned into an external test set. 57 positive and 31 negative images were selected from the training set for the internal validation. Resnet deep learning convolutional network model was utilized for training. Upon training of a self-developed intelligent image-reading system using the two sets, the system read the positive images and negative ones in accordance with 2018 ICM diagnostic indicators and made the diagnosis (Figs. 1, 2 and 3).

1. Training stage: The training stage included data pre-processing, network model construction, network initialization, and training of the model and other modules and components.
  Data pre-processing: This experiment mainly used the technique of horizontal flipping to enhance the data of the training set images, increasing the amount of training data and improving the generalizing ability of the model. The normalization of the image tensor could prevent the saturation of neuron output caused by excessive net input absolute value.
  Network model construction: ResNet34 was employed as the fundamental network structure in this experiment.
  Model training: First, the processed data set was input to the network, the output predictive values and the data truth values were input to the loss function for calculation, and the inverse gradient was input to the optimizer, so as to update the weight of the model. The training was running until the model attained a good learning effect and the model was saved.
2. Test stage: The test stage involved pre-processing, network model prediction and development of other modules.
  Data preprocessing: the test set images do not perform data enhancement operation, but only perform image tensor normalization.
  Test model: The flow of the test model was essentially the same as that of the training model, the differences were as follows: (1) The training process required the calculation of the reverse gradient to update the model weight, but the testing process did not; (2) In the training process, the forward and reverse calculation of each image was done several times. In the test process, only one forward calculation was performed for each image; (3) The ultimate goal of the test process was to judge the correctness of the result and calculate the accuracy of model prediction.

## Results

The model was internally validated by employing the images used for the model establishment for the test of feasibility. 57 positive images and 31 negative images were selected from the aforementioned 461 images. The accuracy for positive images was 96.49%, and the accuracy for the negative ones was 87.09%. The precision rate and the recall rate were 93.22% and 96.49%, respectively, with F1 being 0.9482.

In this experiment, deep learning based on ResNet34 neural network model was used to identify and classify pathological images, and the accuracy of external test set reached 95.22%. The accuracy of external test results was 97.39% for positive images and 93.04% for negative image, respectively, the average accuracy of test being 93.33%. The average recall rate was 97.39%, with an F1 of 0.9532.

For internal verification, the images used for training were employed, and the internal verification result was an initial result when training was half-done. External test result was the result when training was completed with images that had never been used in the training. Table 1 shows that the external test results were better than those of the internal verification, indicating that the convolutional network deep learning improved with training and the diagnostic accuracy increased.

**Table 1:** Validation results

	**Positive accuracy (%)**	**Negative accuracy (%)**
Internal validation set	96.49	87.09
External test set	97.39	93.04

As illustrated in Table 2, the results of the external test set were better than that of the internal validation set on the whole. The external test showed a higher diagnostic accuracy. The convolutional network deep learning method could be further optimized with the accumulation of data of the training set. At the same time, the recall rate of positive images was higher than the accuracy rate, indicating that the algorithm could identify most infections, and the missing rate of infections was low.

**Table 2:** External test results

Positive image	Average accuracy (%)	Average recall rate (%)	F1 index
Internal validation set	93.22	96.49	0.9482
External test set	93.33	97.39	0.9532

**Fig. 4:**
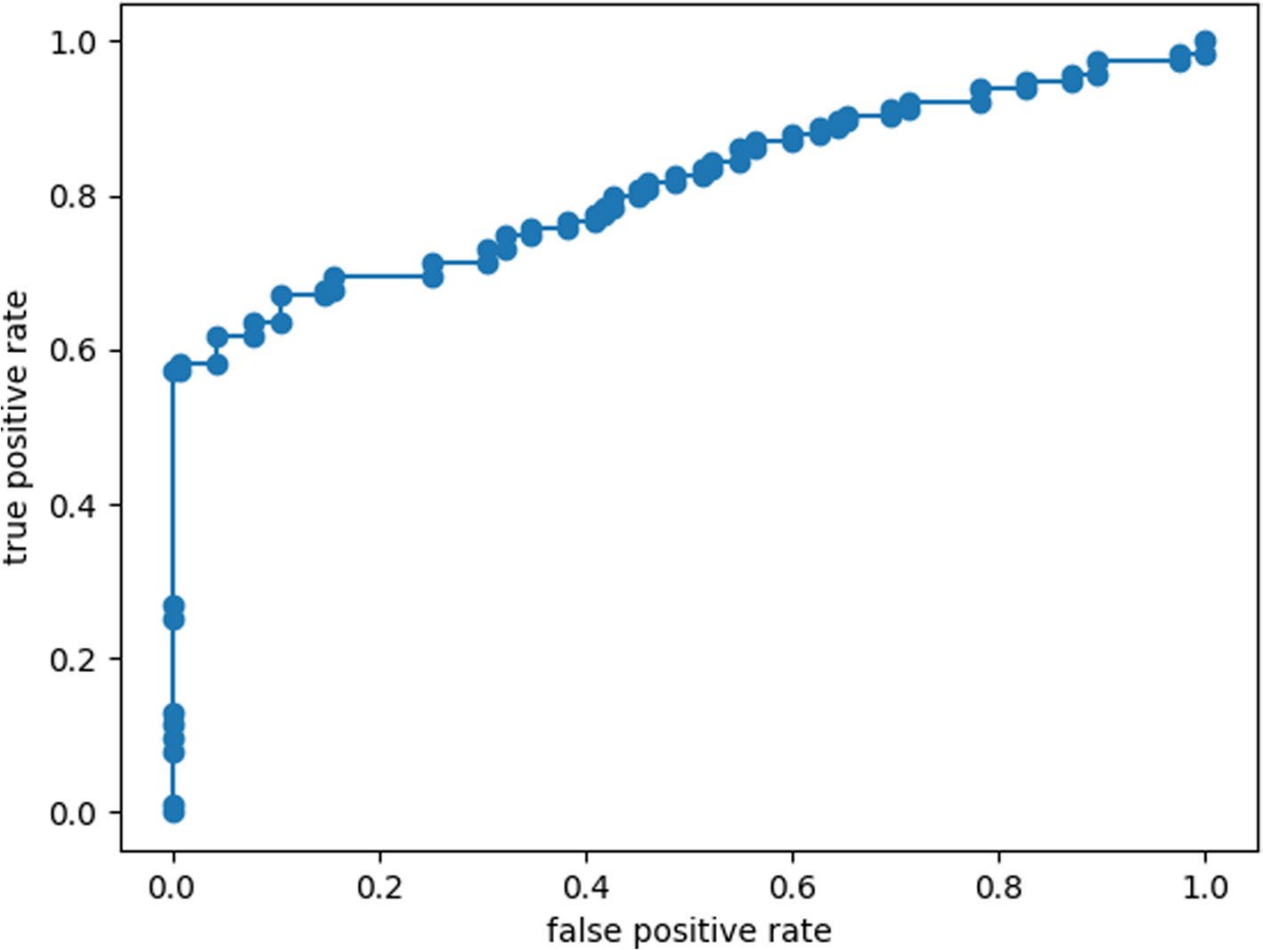
ROC curve of test

The closer to (0, 1) the ROC curve is, the further the point deviates from the 45° diagonal (from the bottom left to the top right), the larger the area under the curve (AUC), indicating that the classifying effect of binary classifier is better (Fig. 4).

An ROC curve corresponding to a binary automatic recognition result is shown in Fig. 4, and AUC area was 0.8136. Statistically, ROC curve is used to represent the discriminating ability of variable threshold binary classifier. The horizontal coordinate of the curve is the false positive rate (FPR) under different thresholds, that is, the proportion of the samples identified to be positive in all negative samples. The ordinate is the true positive rate (TPR), namely, the proportion of the samples that are identified to be positive in all positive samples. The ROC curve in the figure deviates from the diagonal and is closer to point (0, 1), indicating that the model has a good classifying effect.

**Fig. 5:**
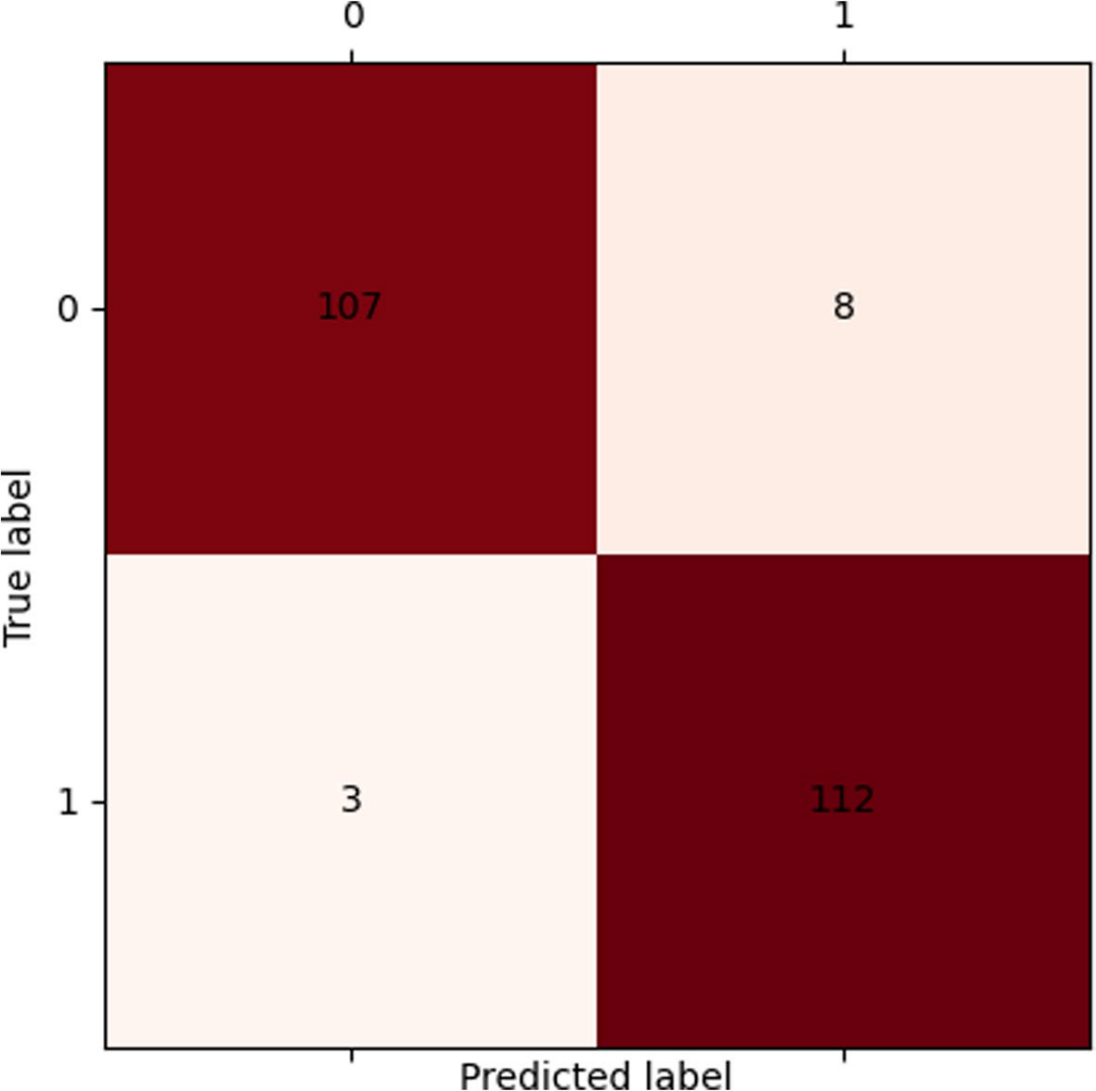
Confusion matrix of test

The Fig. 5 shows that the confusion is not conspicuous in the experimental results, and a good classifying effect was achieved. This marginal confusion could be alleviated by increasing sample size, further enriching annotation data and fine-tuning hyper-parameters.

The corresponding confusion matrix is shown in Fig. 5. The horizontal axis is the infection cases identified; the vertical axis is the cases of infections. “0” represents the negative cases, “1” denotes the positive (infection) cases, and the colors of the grid indicate the presence or absence of infection, with red being infected cases and the yellow the negative cases. The darker the color, the larger the case size. The values outside the diagonals (upper left to lower right) of the confusion matrix are the degree of confusion in corresponding cases. The darker the squares outside the diagonals, the greater the confusion. The figure shows that the confusion was not obvious in the experimental results, and a good classification effect was attained. This slight confusion could be eased by increasing sample size, further enriching annotation data and fine-tuning hyper-parameters.

In this study, the deep learning convolutional network system was used, upon learning, to diagnose PJI and learn to identify the images, positive or negative, under high magnifications, and analyze and synthesize the image features under high magnifications. Comprehensive indicators, such as tissue edema and necrosis of varying severity, texture of infected tissues, infiltration and proliferation of neutrophils and other inflammatory cells and hyperplasia of capillaries were combined to determine whether a given field was positive for infection. Compared with neutrophil count alone, using infection-related findings as basis of pathological diagnosis could accomplish higher sensitivity and specificity.

Through neural network deep learning, we established an intelligent image reading system that could distinguish between the positive and negative images against the 2018 ICM criterion (more than 10 neutrophils per high power field, Table 3). Meanwhile, we preliminarily explored the possibility of comprehensively evaluating the model of infection by means of deep learning network. The infection model integrated tissue edema, necrosis of varying degrees, texture of infected tissues, infiltration of neutrophils and other inflammatory cells and hyperplasia of capillaries in the pathological assessment and diagnosis of images.

**Table 3:**
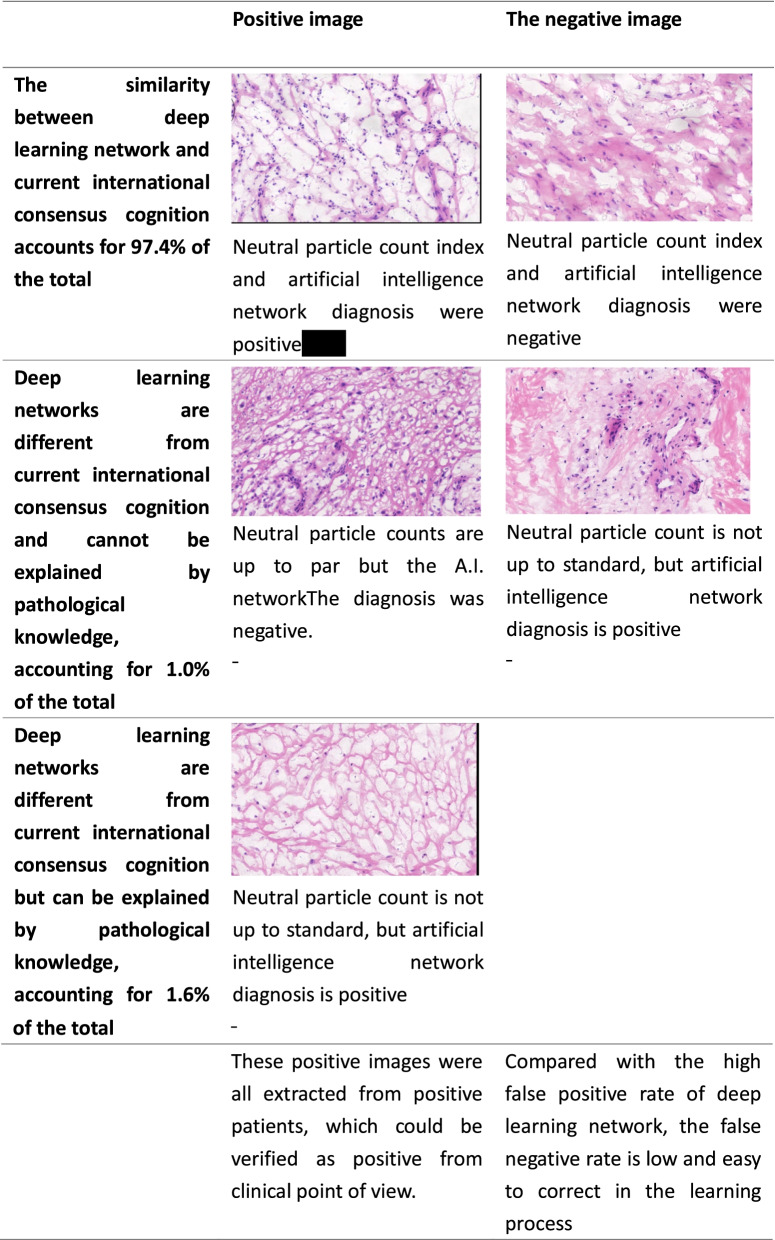
Model case analysis

Basic data of the patients included age, gender, BMI, type of joint replacement procedures and sides operated (Table 4). The patients were divided into two groups in terms of diagnostic results (PJI-positive or PJI-negative). The number assigned to each patient in the table also applies in Table 5.

**Table 4:** Basic data of the patients

Group	*n*	Sex	Age	Height (m)	Weight (Kg)	BMI	Operation
negative	1	female	28	1.50	42	18.67	right hip arthroplasty
	2	female	79	1.60	61.5	24.02	right hip arthroplasty
	3	female	67	1.60	78	30.47	right hip arthroplasty
	4	female	64	1.60	71	27.73	left knee arthroplasty
	5	female	64	1.60	71	27.73	left knee arthroplasty
	6	female	76	1.60	60	23.44	left knee arthroplasty
	7	male	43	1.70	75	25.95	left hip arthroplasty
	8	male	36	1.70	70	24.22	right hip arthroplasty
	9	male	70	1.60	65	25.39	left knee arthroplasty
	10	male	54	1.74	92	30.39	left knee arthroplasty
positive	11	male	80	1.70	70	24.22	left knee arthroplasty
	12	male	30	1.75	95	31.02	left hip arthroplasty
	13	male	60	1.69	65	22.76	left knee arthroplasty
	14	male	85	1.65	73	26.81	left knee arthroplasty
	15	male	53	1.78	80	25.25	right hip arthroplasty
	16	male	23	1.70	95	32.87	right hip arthroplasty
	17	male	59	1.76	80	25.83	left hip arthroplasty
	18	female	67	1.60	78	30.47	right hip arthroplasty
	19	female	67	1.63	72	27.10	right knee arthroplasty
	20	female	68	1.50	54	24.00	left hip arthroplasty

**Table 5:** Patient diagnosis relevant data

Group + AA1:J26	*n*	WBC positive	PMN%	CRP	IL-6	ESR	D-dimmer	Bacterial culture results
negative	1	-	-	-	-	-	-	-
	2	-	-	-	+	-	-	-
	3	-	+	-	-	-	-	-
	4	-	-	-	-	-	-	-
	5	-	-	-	+	-	-	-
	6	-	-	-	-	-	-	-
	7	-	–	-	-	-	-	-
	8	-	–	-	-	-	-	-
	9	-	-	+	-	-	-	-
	10	-	-	-	-	+	-	-
positive	11	+	+	-	+	+	+	*Staphylococcus aureus*
	12	+	+ +	+	+ +	+	-	*Staphylococcus epidermidis*
	13	-	-	+	+ +	+	+	*Staphylococcus epidermidis*
	14	+	+	+	+ +	+	+	*Staphylococcus epidermidis*
	15	+	+	+ +	-	+	+ +	*Streptococcus pharyngitis*
	16	-	-	+	+ +	+	+	*Klebsiella pneumoniae*
	17	-	+	+ +	+ +	+	-	Gram-positive bacilli
	18	-	+	+	+ +	+	+	*Staphylococcus aureus*
	19	+	+	-	+	+	+	*Propionibacterium bullosa*
	20	+	+ +	+ +	+ +	+	+	*Staphylococcus aureus*
		4–10 -	< 0.5 –	< 2 -	0–5.9 -	0–20 -	0–0.55 -	
		11–20 +	0.5–0.7 -	2–10 +	5.9–23.6 +	> 20 +	0.55–5.5 +	
		> 20 + +	0.7–0.9 +	> 10 + +	> 23.6 + +			
			> 0.9 + +					

The patient was diagnosed with PJI according to the 2018 ICM diagnostic criteria, which specify that normally the patient's white blood cell count (WBC) ranges from 3.5 × 10^8^/L to 10 × 10^8^/L, the neutrophil percentage (PMN) from 0.5 to 0.7, C-reactive protein (CRP) from 0–0.8 mg/dL, interleukin-6 (IL-6) from 0 to 5.9 pg/mL, the erythrocyte sedimentation rate (ESR) from 0 to 20 mm/h, the plasma D-dimer from 0 to 0.55 µg/mL. The infection-related bacteria were identified by culture of joint fluid extract harvested preoperatively or tissues collected intraoperatively and the identification result was further confirmed if the bacteria of the two kinds of samples were identical.

## Discussion

PJI represents a common and catastrophic complication after artificial joint replacement [1] and is estimated to occur in 1–3% of patients undergoing primary replacement and in 3–5% of patients undergoing revision [11]. With the improvement and increasingly wider application of the joint replacement surgery and related technologies, more and more patients receive the procedure. The incidences of PJI have been on the rise over the recent years and accurate diagnosis of the complication is an urgent task of clinical research.

The current international consensus regarding the pathological diagnosis of PJI is the result of a protracted research endeavor. PJI is pathologically characterized by acute infectious inflammation of soft tissues, and has been described in terms of neutrophil infiltration, tissue edema, and necrosis of varying degrees and hyperplasia of capillaries [17]. Current diagnostic criteria are based on the feature of neutrophils, alone, to distinguish it from other infections in a simplified and standardized way. When neutrophils were initially used as a measure of pathological diagnosis, a field with over 23 neutrophils under 400 × magnifications were taken as a positive area. Nonetheless, the specificity of this diagnostic index was merely 23% [18]. Subsequently, a series of international conferences modified this standard, and the most recent ICM consensus reached in 2018 proposed that a high magnification field containing more than 10 neutrophils was deemed as an area positive for infection [11]. According to UJBIS consensus in 2021, a 400 × field of vision with more than 5 neutrophils was seen as a positive area [19]. The pathological diagnosis of PJI has been constantly evolving towards standardization and higher accuracy, but has been hindered by such problems as long time and low specificity, among others.

In recent years, medical researchers are increasingly employing network deep learning and achieved good results. Rajpurkar et al. used AI to screen lung cancer from chest radiographs [20] and attained impressive results. Although a great many difficulties remain in the clinical application of AI to the diagnosis of CT or MRI or other complicated images, use of AI algorithms in the diagnosis of microscopic images with few layers and relatively simple patterns can attain better results. Bang et al. reviewed 8 studies and concluded that AI algorithm could be used as a reliable tool for endoscopic diagnosis of *Helicobacter pylori* infection [21]. This review suggests that it is possible to use AI algorithm as an auxiliary tool in the diagnosis and treatment. The application of convolutional networks in the reading of pathological sections were not uncommon. Hermsen et al. trained a convolutional neural network [22] for multi-class segmentation of digital renal tissue sections and it was able to identify glomeruli, renal tubules and interstitia in digitalized renal tissue sections. Han et al. also used convolutional neural network trained from Asan dataset, Med-Node dataset and Atlas site images to classify images of 12 different skin conditions, and achieved a sensitivity comparable to that attained by dermatologists in identifying basal cell carcinoma [23]. The above two experiments suggest that convolutional network learning is feasible in identifying some characteristic regions, such as the region of kidney or regions where cancer cells gather. So far, studies are scanty on the use of the convolutional network learning in the pathological diagnosis of inflammation and infection. Han et al. trained region-based convolutional neural network to diagnose fungal infection of lichenoid lesions and the network outperformed dermatologists [24].

In the past, pathologists have empirically put forward some features of the infected area but the features are hard to describe or no uniform or standardized identification methods are available. The pathology of infection has been well studied, mainly in tissues of epithelial and acinar cells, including pulmonary, gastric mucosal and vaginal tissues, which are histopathologically highly specific. For instance, a study on lung infection by Hussain et al. showed that the infected lungs had a localized centrally-calcified necrotic area, with chronic infiltration of inflammatory cells into the margins [25]. However, pathological identification of deep tissues, in general, including connective tissue in particular, is difficult because the aforementioned characteristics may not be applicable to non-mucosal tissues. In 2009, Krenn et al. proposed [18] that in aseptic loosening and infection-related loosening of prosthesis, a peri-prosthetic membrane exists around the prosthesis, and the cause of loosening can be distinguished by the pathological differences of the membrane. Although there are many pathological differences in the membrane, the most convenient and reproducible measure is 23 neutrophils in 10 high power fields (HPFs). Sigmund et al. applied this diagnostic criterion to the frozen sections and proved that the result was no different from that of permanent sections [11]. According to the diagnostic criteria formulated at the ICM in 2018, pathological diagnosis can be definitively made if more than 10 neutrophils are found in 5 fields selected from multiple high-magnification ones [10]. This is in contrast with the criterion put forth in 2021 (UJBIS), which states that more than 5 neutrophils found in the 5 high magnification fields can establish pathological diagnosis [19].

Though international organizations have been relentlessly improving the diagnostic accuracy by revising the number of neutrophils, traditional (human) diagnostic approaches are still fraught with the tough problems of being time-consuming and having low sensitivity [11]. First of all, artificial identification of infected areas often fails to cover the entire pathological section area, which tends to cause the omission of infected areas. Meanwhile, neutrophils have various forms and are easy to be confused with other inflammatory cells, resulting in counting deviation. Second, artificial chemical staining and artificial counting of white blood cells are time-consuming and costly [12], rendering it difficult to make timely intraoperative diagnosis on the basis of pathological specimens. Ultimately, pathological diagnosis of PJI is not sensitive. The low sensitivity results not only from omission of infected fields on the sections but also from the inadequate understanding about neutrophil morphology. Moreover, the very use of neutrophils as an indicator also presents some problems. Admittedly, the new diagnostic criterion, to some degrees, enhances the specificity of the diagnosis (up to about 50%) when maintaining its sensitivity at an appropriate level [10]. The UJBIS consensus takes a 400 × field of vision with more than 5 neutrophils as an area positive for infection, and the specificity is too low to make it a major diagnostic measure [19].

In this experiment, a batch of images were selected as the training set against the existing ICM diagnostic criteria (more than 10 neutrophils in a 400 × field). After external verification, the intelligent diagnosis system registered a high positive recognition rate and a high negative recognition rate. Our analysis revealed that the artificial intelligence system did not identify infected areas in terms of neutrophils alone and it also made diagnosis by comprehensively assessing tissue edema, necrosis, tissue texture, infiltration of neutrophils and other inflammatory cells, and proliferation of capillaries, among others. This comprehensive diagnostic approach deserves further studies since it achieved a diagnostic sensitivity comparable to the artificial judgment of similar images under the high-power field.

The convolutional network deep learning has the advantages of high throughput, good reproducibility and high accuracy for the identification of infected regions of PJI images at high magnifications. Compared to the linear nature of manual recognition, convolutional network deep learning can simultaneously analyze multiple images. In the process of revision surgery, the time saved may mean lowered surgical risk and more favorable prognosis for patients [26]. On the other hand, artificial (human) recognition of pathological images is highly subjective, and results might vary with different readers/pathologists. The results of image reading by convolutional network deep learning are repeatable, and are therefore evidence of higher levels. Additionally, for human reading of pathological sections, it is often necessary to select highly infected areas first, and then the high-magnification fields were scanned, which inevitably results in the omission of infected areas and the misjudgment about neutrophils. However, convolutional network deep learning can cover the entire section, without omission, thereby achieving more accurate diagnosis.

Pathologically, infection of the adjacent tissues of a joint manifests as acute inflammation. Under a microscope, it presents more than the accumulation of neutrophils to include soft tissue edema, muscular necrosis of different degrees, infiltration of a variety of inflammatory cells, even changes in the tissue internal environment [18]. The features are sensitive but not specific and are difficult to be translated into quantitative criteria. With the convolutional network deep learning, all the details of acute inflammation in an image can be enhanced and quantified and the resultant images can serve as data for model training or image recognition. Use of convolutional network deep learning not only avoids the judgment of the complex morphology of neutrophils and but also gets around the age-old controversy over the number of neutrophils.

The internal verification utilized the images that had been used for training, and the internal verification result was an initial result when training was half-done. For external test, the images that had not been used in the training were employed, and the external result was the ultimate result when training was completed. Tables 1 and 2 show that the external test results were better than those of the internal verification, indicating that the convolutional network deep learning improved with training and the diagnostic accuracy also increased.

Our preliminary application of artificial intelligence in the pathological diagnosis of PJI is a new attempt and some limitations are obvious. First of all, we adopted retrospective analysis to select the sample population, and the basic sample size of section was small, which may have caused omission of some pathological characteristics of PJI. Second, the diagnostic results of this deep learning convolutional network were still different from the results based on the expert consensus of 2018 ICM [12]. Better results can be achieved by comparing the new method with the standard strategies in terms of diagnostic accuracy. Our method integrated such features as histological characteristics of infection (edema, necrosis and accumulation of neutraphils) and the overall morphology of the section (grains, color and pixel size) into the assessment of the images. Continuous upgrading of extended training sets is needed to improve the diagnostic accuracy of the convolutional network deep learning before it is applied to clinical practice.

## Conclusion

In this study, resNET model, a deep learning algorithm, was trained to identify high-magnification images from pathological sections of soft tissues around joints, against the diagnostic criteria for acute infection, and a high precision and a high recall rate were accomplished. This technique may improve the accuracy of pathological diagnosis of PJI by more comprehensively assessing the pathological features of PJI. Further studies are needed to determine the feasibility of these results in prospective clinical trials.

## Data Availability

The datasets used and/or analyzed during the current study are available from corresponding author on reasonable request.
